# Selective reporting in clinical trials - an examination of discrepancy rates in pre-specified and reported outcomes in articles submitted to the BMJ

**DOI:** 10.1186/1745-6215-16-S2-O72

**Published:** 2015-11-16

**Authors:** Jennifer Weston, Kerry Dwan, Douglas Altman, Mike Clarke, Carrol Gamble, Trish Groves, Sara Schroter, Paula Williamson, Jamie Kirkham

**Affiliations:** 1 University of Liverpool, Liverpool, UK; 2 University of Oxford, Oxford, UK; 3 Queen's University Belfast, Belfast, UK; 4 The BMJ, London, UK

## Background

Adding, omitting or changing pre-specified outcomes can result in bias because it increases the potential for unacknowledged or post-hoc revisions of the planned analyses. Journals have adopted initiatives such as requiring the prospective registration of trials to promote the transparency of reporting in clinical trials.

## Methods

A review of all 3156 articles submitted to The BMJ between September 2013 and July 2014. Trial registry entries, protocols and trial reports of randomised controlled trials published by The BMJ and a random sample of those rejected were reviewed to determine the frequency and type of outcome discrepancies between pre-specified and reported outcomes. Editorial, peer reviewer comments and author responses were also examined to ascertain any reasons for discrepancies.

## Results

In the study period, The BMJ received 311 trial manuscripts, 21 of which were published by the journal. In trials published by The BMJ, 22% (75/339) of the pre-specified outcomes were not reported and 8% (25/297) of reported outcomes were not pre-specified. Discrepancy rates for rejected articles were similar. The majority of reasons provided by authors for outcome discrepancies were not bias related.

## Conclusions

Mandating the prospective registration of a trial and requesting that a protocol be uploaded when submitting a trial article to a journal has the potential to promote transparency and safeguard the evidence base against outcome reporting biases as a result of outcome discrepancies. Further guidance is needed with regards to documenting reasons for outcome discrepancies and making these reasons available.

